# Do Medical Students Adopt Problem-Focused or Emotion-Focused Coping Strategies?

**DOI:** 10.1192/bjo.2024.160

**Published:** 2024-08-01

**Authors:** Aisha Hawsawi, Elena Nixon, Neil Nixon

**Affiliations:** University of Nottingham, Nottingham, United Kingdom

## Abstract

**Aims:**

The pursuit of a career in medicine, while potentially rewarding, is undeniably accompanied by demanding challenges. These challenges encompass not only rigorous academic demands and long work hours but also contend with a competitive academic environment, conflicts in maintaining a study-life balance, and a multitude of other stressors unique to the medical profession. Amidst this backdrop, concerns are growing worldwide about the mental health challenges that medical students face as they start their careers in medicine. Coping can play a pivotal role in overcoming these challenges. This study explores how coping is associated with wellbeing aspects, i.e., anxiety and depression, as well as personality, and looks into the coping strategies adopted by medical students, specifically focusing on whether they predominantly employ problem-focused or emotion-focused coping. Additionally, it aims to explore contextual factors influencing students' coping strategies, which is crucial for informing wellbeing interventions and support services.

**Methods:**

This study used a mixed-methods approach, employing quantitative data on coping, personality, stress, anxiety and depression and qualitative data from semi-structured interviews with preclinical and clinical year medical students at the University of Nottingham.

**Results:**

Regression findings revealed that medical students primarily used emotion-focused over problem-focused coping. Interestingly, thematic analysis showed that medical students employ problem-focused coping strategies in rigorous, academically challenging and controllable situations such as upcoming exams; they prioritise structured study schedules, seek additional academic resources, and actively engage with faculty to enhance their understanding of complex topics; conversely, emotion-focused coping emerged prominently in the face of personal or interpersonal stressors, particularly in situations perceived as uncontrollable. In such instances, like unexpected setbacks or health concerns, students may acknowledge and express their emotions and engage in activities for emotional relief, including seeking wellbeing support.

**Conclusion:**

The study reveals a dynamic interplay between problem-focused and emotion-focused coping strategies in medical students. Recognising that medical students tend to adopt different coping strategies in different situations, medical education systems should aim to develop or tailor existing resources to provide appropriate academic and wellbeing support.

